# Beyond the guidelines: Vascular function as the central determinant and therapeutic target in hypertension

**DOI:** 10.1038/s41440-026-02762-x

**Published:** 2026-08-04

**Authors:** Yukihito Higashi

**Affiliations:** 1 Department of Regenerative Medicine, Division of Radiation Medical Science, Research Institute for Radiation Biology and Medicine, Hiroshima University, Hiroshima, Japan; 2 Division of Regeneration and Medicine, Hiroshima University Hospital, Hiroshima, Japan

**Keywords:** Hypertension, Vascular function, Endothelial function, Nitric oxide

## Abstract

Hypertension has traditionally been defined and managed according to brachial blood pressure levels. Although this pressure-centric strategy has significantly reduced cardiovascular morbidity and mortality, substantial residual risk persists even among patients who achieve recommended blood pressure targets. Emerging evidence indicates that hypertension is fundamentally a systemic disorder of the vascular wall, in which endothelial dysfunction, oxidative stress, immune activation, microvascular remodeling, and progressive arterial stiffening not only result from elevated pressure but also actively drive its initiation and progression. Endothelial nitric oxide deficiency, redox imbalance with endothelial nitric oxide synthase uncoupling, T helper 17 cell-mediated inflammation, and mineralocorticoid receptor–dependent vascular signaling collectively promote increased vascular tone and structural remodeling. Microvascular rarefaction elevates peripheral resistance, while large-artery stiffening augments central pulsatile load and accelerates target organ injury. These interconnected processes create a self-reinforcing network that sustains hypertension beyond simple hemodynamic elevation. In this review, I propose a shift from a pressure-centric to a vessel-centric paradigm of hypertension. I describe mechanistically defined vascular phenotypes, discuss macrovascular and microvascular assessment strategies, and explore therapeutic approaches aimed at vascular restoration in addition to blood pressure reduction. I further examine the concepts of vascular age, residual risk, and precision vascular medicine, and consider future integration of artificial intelligence and vascular endpoints into clinical trials. Recognizing vascular dysfunction as a central determinant of hypertension may refine risk stratification, redefine therapeutic success, and represent the next frontier in cardiovascular medicine.

**Figure:**
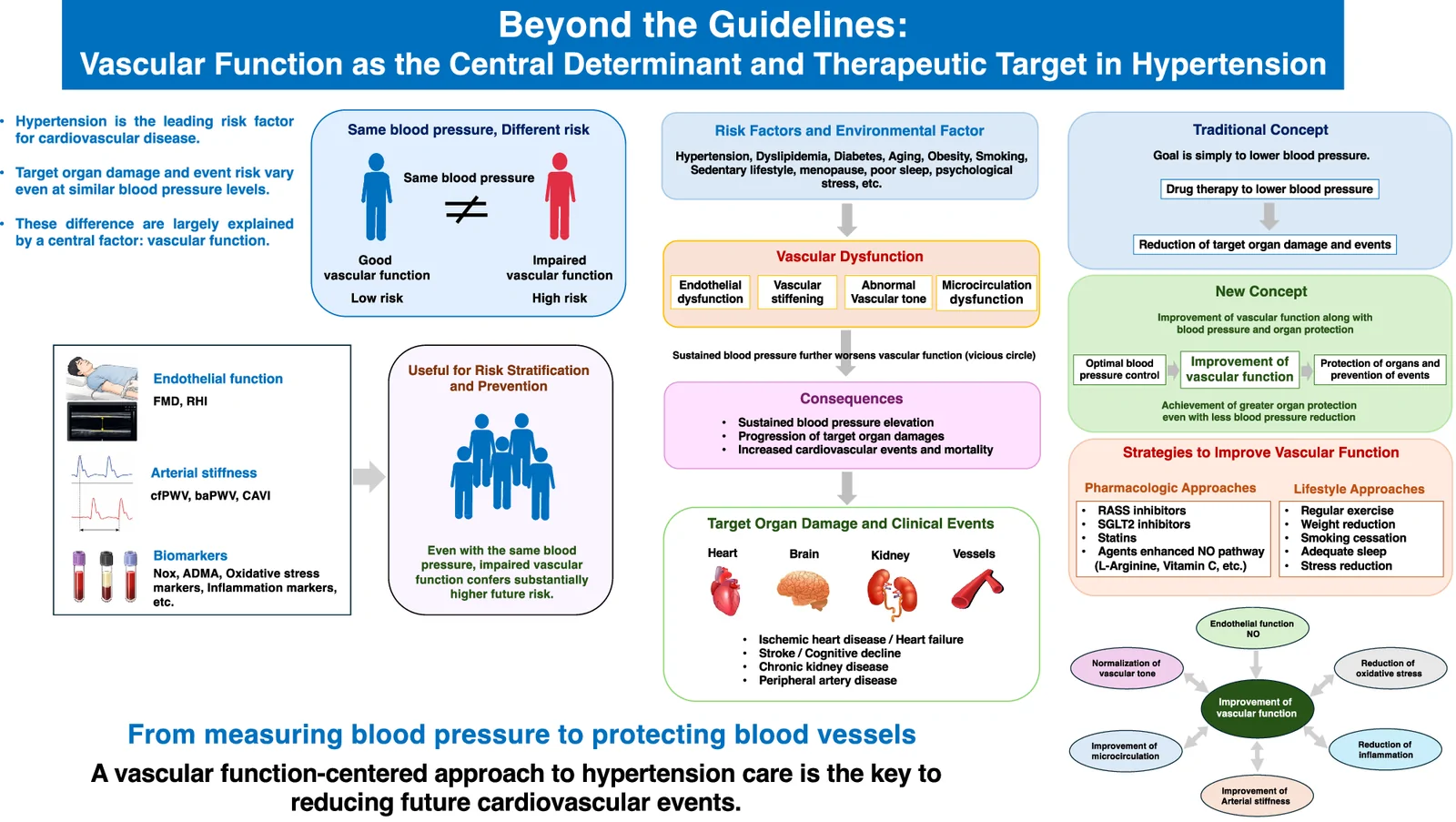
Beyond Blood Pressure: Vascular Function as the Central Determinant and Therapeutic Target in Hypertension. FMD indicates flow-mediated vasodilation, RHI reactive hyperemia index, cFPWV carotid-femoral pulse wave velocity, baPWV brachial-ankle PWV, CAVI cardio ankle vascular index, Nox nitrite/nitrate, ADMA asymmeteric dimethylarginen, RAAS renin-angiotensin-aldosterone system, SGLT2 sodium–glucose cotransporter 2, NO nitric oxide

Beyond Blood Pressure: Vascular Function as the Central Determinant and Therapeutic Target in Hypertension. FMD indicates flow-mediated vasodilation, RHI reactive hyperemia index, cFPWV carotid-femoral pulse wave velocity, baPWV brachial-ankle PWV, CAVI cardio ankle vascular index, Nox nitrite/nitrate, ADMA asymmeteric dimethylarginen, RAAS renin-angiotensin-aldosterone system, SGLT2 sodium–glucose cotransporter 2, NO nitric oxide

## Abstract (web summary)

Beyond Blood Pressure: Vascular Function as the Central Determinant and Therapeutic Target in Hypertension. FMD indicates flow-mediated vasodilation; RHI, reactive hyperemia index; cFPWV, carotid-femoral pulse wave velocity; baPWV, brachial-ankle PWV; CAVI, cardio ankle vascular index; Nox, nitrite/nitrate; ADMA, asymmeteric dimethylarginen; RAAS, renin-angiotensin-aldosterone system; SGLT2, sodium–glucose cotransporter 2; NO, nitric oxide.

## Introduction

Hypertension remains the leading modifiable risk factor for cardiovascular morbidity and mortality worldwide [1–4]. Contemporary management strategies, shaped by major international guidelines, have focused primarily on achieving and maintaining target blood pressure levels [1–4]. This pressure-centric paradigm has undoubtedly reduced cardiovascular events at the population level. However, a substantial residual risk persists even among individuals whose clinic and ambulatory blood pressure values are within recommended ranges [5, 6]. This observation suggests that blood pressure reduction alone may not fully address the underlying vascular pathology that drives cardiovascular disease.

Increasing experimental and clinical evidence indicates that hypertension is not merely a hemodynamic disorder but fundamentally a disease of the vascular wall [7, 8]. Endothelial dysfunction represents one of the earliest detectable abnormalities in individuals with elevated blood pressure and even in those with high-normal blood pressure [9–12]. Impairment of nitric oxide (NO) bioavailability increased oxidative stress, endothelial NO synthase (eNOS) uncoupling, and inflammatory signaling contribute to a pro-constrictive, pro-thrombotic, and pro-inflammatory vascular milieu [13–18]. Importantly, these alterations are not only consequences of elevated pressure but also active participants in the initiation and maintenance of hypertension [7, 8].

Beyond endothelial dysfunction, structural and functional changes in the microcirculation play a critical role. Microvascular rarefaction, characterized by a reduction in capillary density, increases peripheral vascular resistance and amplifies blood pressure responses to physiological stress [19–24]. Concurrently, large conduit arteries undergo remodeling and progressive stiffening [25–39]. Increased arterial stiffness augments systolic blood pressure, enhances wave reflection, and elevates pulsatile load on the heart, brain, and kidneys [29–33]. These changes establish a self-perpetuating cycle in which vascular injury promotes hypertension, and hypertension further accelerates vascular damage.

Recent advances have further highlighted the role of immune activation and local mineralocorticoid receptor signaling in vascular dysfunction [40–57]. High-sodium environments, metabolic stress, and aging modulate immune responses, promoting pro-inflammatory T-cell activation and cytokine production [42–49]. This immunovascular axis contributes to endothelial injury and vascular remodeling independently of systemic renin–angiotensin–aldosterone system activation [40–57]. Such findings challenge the traditional view that hypertension is solely driven by neurohumoral and renal mechanisms and instead position the vasculature at the center of disease pathogenesis.

Despite these insights, vascular function assessment has not yet been fully integrated into routine hypertension management. Techniques such as flow-mediated dilation (FMD), pulse wave velocity (PWV), cardio-ankle vascular index (CAVI), and microvascular imaging provide mechanistic information beyond brachial blood pressure measurement [58–68]. However, their clinical application remains limited, and vascular endpoints are rarely incorporated into large-scale hypertension trials [25–39, 58–60]. As a result, therapeutic strategies are often evaluated predominantly by their ability to lower blood pressure, rather than by their capacity to restore vascular health.

**Fig. 1:**
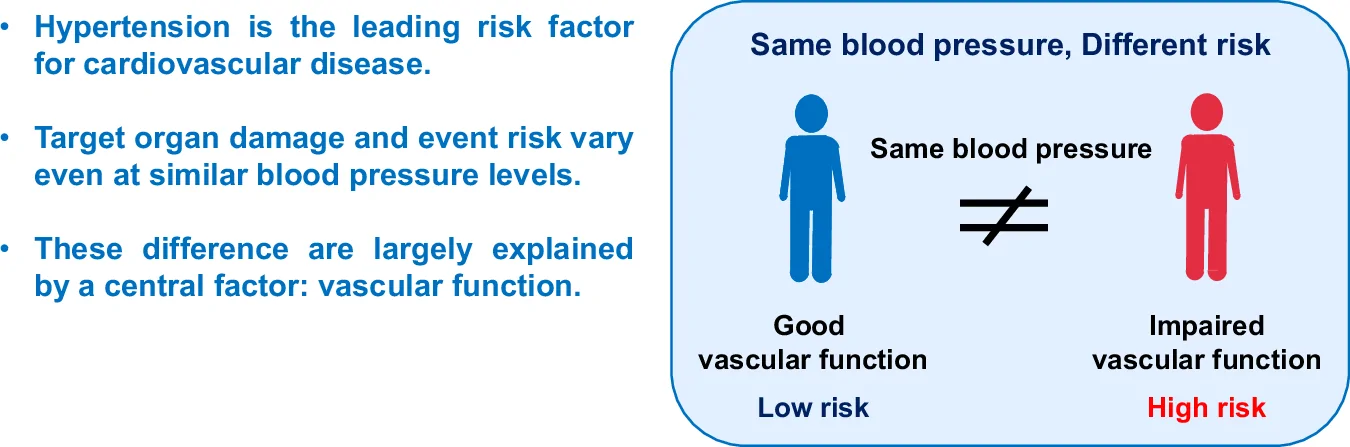
Individual Differences in Targe Organ Damage and Adverse Cardiovascular Events Occur Even at Similar Blood Pressure Levels, Largely Due to Variations in Vascular Function

In this review, I propose a conceptual shift from a pressure-centric to a vessel-centric model of hypertension (Fig. 1). We examine the pathophysiological basis of vascular dysfunction in hypertension, describe distinct vascular phenotypes that may underlie clinical heterogeneity, and explore how vascular assessment could refine risk stratification and therapeutic decision-making. I further discuss the implications of targeting vascular biology as a primary therapeutic objective and outline future directions for precision vascular medicine. Recognizing vascular function as a central determinant of hypertension may redefine how we diagnose, treat, and ultimately prevent hypertensive cardiovascular disease.

## Hypertension as a disease of the vascular wall

### Endothelial dysfunction

**Fig. 2:**
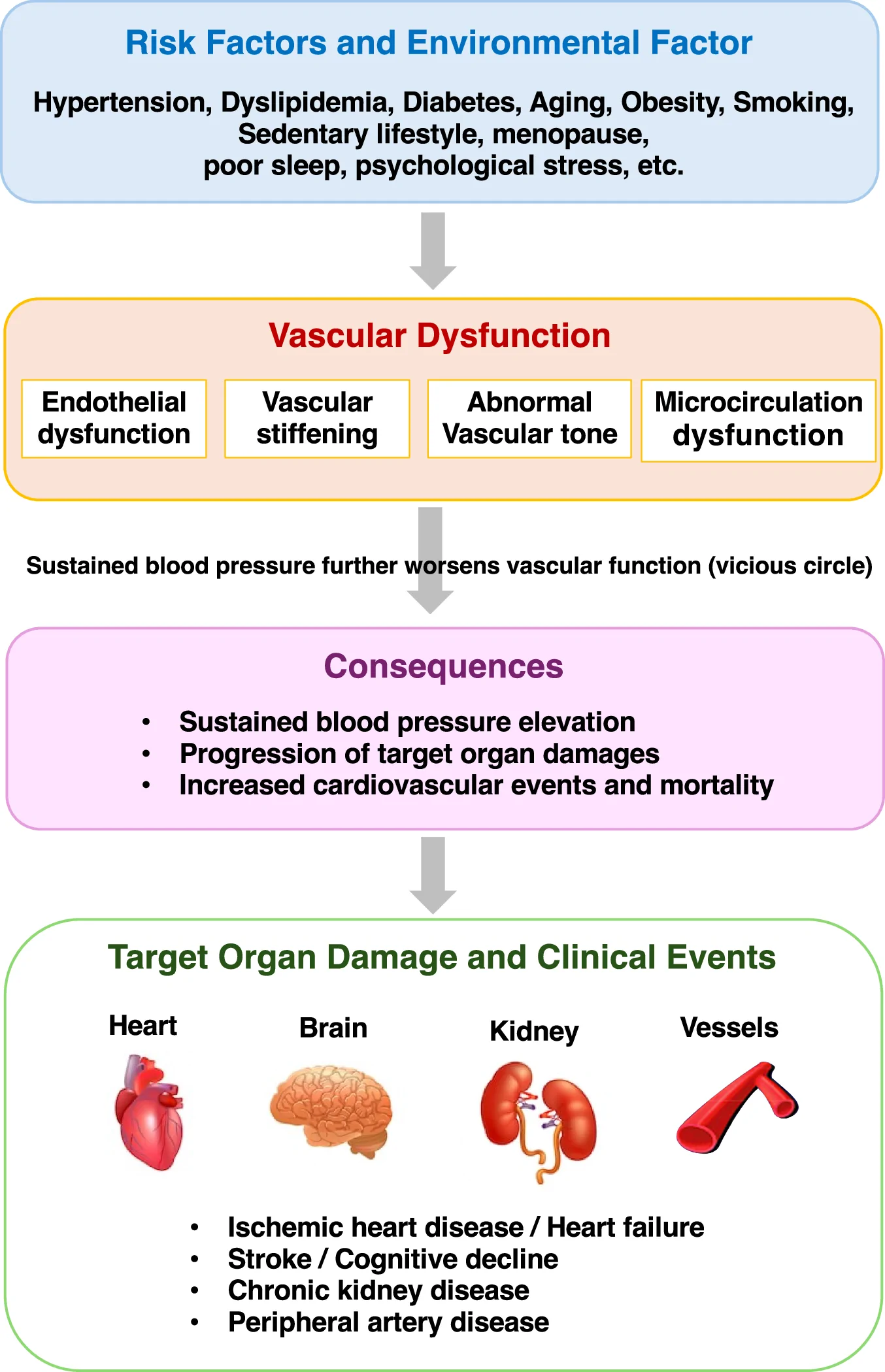
Vascular dysfunction at the core of hypertension

Endothelial dysfunction represents one of the earliest detectable abnormalities in individuals with elevated blood pressure and even in normotensive individuals at increased cardiovascular risk (Fig. 2) [9–12]. Rather than being a passive consequence of mechanical stress, endothelial dysfunction is increasingly recognized as a primary pathophysiological driver of hypertension [7, 8]. Under physiological conditions, eNOS generates NO, which maintains vascular homeostasis by promoting vasodilation, inhibiting platelet aggregation, suppressing leukocyte adhesion, and restraining smooth muscle proliferation [13, 69–72]. In hypertension, NO bioavailability is reduced through multiple mechanisms, including diminished eNOS expression, impaired phosphorylation at activating sites (e.g., Ser1177), and increased degradation of NO by reactive oxygen species (ROS) [16, 73, 74]. Importantly, eNOS uncoupling represents a critical mechanistic shift. In the setting of tetrahydrobiopterin (BH_4_) oxidation or L-arginine deficiency, eNOS becomes a source of superoxide rather than NO, transforming a vasoprotective enzyme into a pro-oxidant mediator [74–76]. This transition amplifies oxidative stress and perpetuates endothelial dysfunction. Endothelial sodium channel (ENaC) activation under high-sodium conditions further alters endothelial biomechanics [53, 54]. Increased intracellular sodium promotes cortical actin polymerization, enhancing endothelial stiffness and impairing shear stress–induced NO release [53, 54]. In parallel, degradation of the endothelial glycocalyx reduces sodium buffering capacity and disrupts mechanotransduction [77–81]. These structural changes impair FND even before sustained blood pressure elevation develops. Clinical studies demonstrate that impaired FMD predicts incident hypertension and cardiovascular events, supporting the concept that endothelial dysfunction is not merely secondary to hypertension but may precede and contribute to its development [55–64, 81–87]. Endothelial injury therefore functions as a mechanistic bridge linking metabolic, inflammatory, and hemodynamic stressors to sustained blood pressure elevation.

### Oxidative stress and NO signaling

Oxidative stress is a central integrator of vascular dysfunction in hypertension [25, 76, 88–100]. The predominant sources of vascular ROS include nicotinamide adenine dinucleotide phosphate (NADPH) oxidases (particularly NOx2 and NOx4), dysfunctional mitochondria, xanthine oxidase, and uncoupled eNOS [75, 88–96]. Angiotensin II (Ang II), aldosterone, mechanical stretch, and inflammatory cytokines stimulate NOx activation via protein kinase C–dependent and Rac1-mediated pathways [75, 91–94]. Superoxide generated by NOx rapidly reacts with NO to form peroxynitrite, which nitrates tyrosine residues in proteins and oxidizes BH_4_, thereby promoting further eNOS uncoupling [98–100]. This establishes a feed-forward redox loop in which oxidative stress both reduces NO bioavailability and enhances ROS production [75, 76, 88–100]. Mitochondrial dysfunction further contributes to redox imbalance [90, 95]. Increased mitochondrial ROS production activates redox-sensitive transcription factors such as nuclear factor-κB and activating protein-1, upregulating pro-inflammatory and pro-fibrotic gene expression [93, 96]. Oxidative stress also activates matrix metalloproteinases, facilitating extracellular matrix remodeling and elastin fragmentation, thereby contributing to arterial stiffening [36, 37]. Notably, mechanical stretch induced by elevated blood pressure increases ROS generation through integrin-mediated signaling and activation of redox-sensitive kinases. Thus, oxidative stress is both a cause and a consequence of hypertension, reinforcing a self-perpetuating cycle of vascular injury.

### Immune–vascular interactions

Hypertension is increasingly recognized as a state of chronic low-grade vascular inflammation [40–49]. Experimental studies demonstrate that adaptive immune cells, particularly T lymphocytes, are required for the full development of Ang II–induced hypertension [40, 41]. High-salt environments enhance serum- and glucocorticoid-regulated kinase 1 activity in T cells, promoting differentiation toward the T helper 17 (Th17) phenotype [42, 43]. Interleukin-17 (IL-17) impairs endothelial NO production, increases oxidative stress, and upregulates adhesion molecules such as vascular cell adhesion molecule-1 and intercellular adhesion molecule-1, facilitating leukocyte recruitment into the vascular wall [44, 45]. These immune responses contribute to endothelial dysfunction and vascular remodeling independently of systemic renin–angiotensin–aldosterone system (RAAS) activity [44–49]. Local mineralocorticoid receptor (MR) activation within endothelial and vascular smooth muscle cells further amplifies inflammatory signaling [50–57]. MR activation enhances NADPH oxidase expression, increases collagen synthesis, and promotes fibrosis [50–52]. Importantly, MR signaling within the vascular wall may remain active even when circulating RAAS activity is suppressed by high salt intake, suggesting tissue-specific dissociation between systemic and local pathways [50–57]. The interaction between immune activation, redox imbalance, and endothelial dysfunction establishes an immunovascular axis that contributes to salt sensitivity, vascular stiffness, and sustained hypertension.

### Microvascular rarefaction

Microvascular rarefaction—defined as a reduction in the density of perfused capillaries—represents a critical structural alteration in hypertension [19–24, 101–104]. Both structural rarefaction (loss of capillary segments) and functional rarefaction (impaired capillary recruitment) have been documented [19–24, 101, 102]. Reduced angiogenic signaling, including decreased vascular endothelial growth factor activity, contributes to capillary dropout. Endothelial apoptosis and oxidative stress further exacerbate microvascular loss [19–24, 101–104]. Functionally, increased precapillary arteriolar tone and enhanced endothelin-1 (ET-1) signaling reduce microvascular perfusion even in the absence of structural loss. Rarefaction increases total peripheral resistance by reducing parallel microvascular channels, thereby amplifying systemic vascular resistance [19–24]. Additionally, impaired microcirculatory perfusion promotes tissue hypoxia, which may activate hypoxia-inducible pathways and stimulate further vasoconstrictive and fibrotic signaling [101–104]. Microvascular dysfunction also contributes directly to target organ damage. In the kidney, rarefaction accelerates glomerulosclerosis; in the brain, it promotes white matter injury; and in the heart, it impairs coronary microvascular reserve [101–104]. These findings underscore that hypertension is not confined to large-vessel pathology but involves profound microcirculatory remodeling.

### Arterial stiffness and pulsatile load

Arterial stiffness reflects both structural remodeling and functional alterations in vascular tone [25–39]. Chronic hypertension promotes increased collagen deposition, elastin fragmentation, and advanced glycation end-product crosslinking within the extracellular matrix [36, 37]. Oxidative stress and inflammation accelerate these processes by activating transforming growth factor-β and matrix metalloproteinases [36, 37, 93]. In addition to structural changes, functional stiffness arises from enhanced vascular smooth muscle tone mediated by rhoA/rho-kinase signaling and calcium sensitization pathways [105, 106]. These mechanisms increase arterial wall tension independently of structural fibrosis. Increased PWV augments central systolic blood pressure and enhances wave reflection, increasing left ventricular afterload and myocardial oxygen demand [25–35]. Moreover, excessive pulsatile energy is transmitted to microvascular beds in high-flow organs such as the brain and kidney, contributing to microvascular injury and end-organ damage [29–33]. Thus, vascular remodeling across the arterial tree—from microvessels to large conduit arteries—forms a pathophysiological continuum. Structural and functional alterations reinforce each other, sustaining hypertensive disease even when brachial blood pressure appears controlled.

## Vascular phenotypes of hypertension

Hypertension is not a uniform hemodynamic abnormality but a biologically heterogeneous syndrome arising from distinct yet interacting vascular mechanism [7, 8, 41]. A vessel-centric framework allows classification of hypertension into mechanistically defined vascular phenotypes that reflect the dominant pathobiological processes within the vascular wall [7–9, 107]. These phenotypes are not rigid categories but represent prevailing biological axes that may shift over time as disease progresses [107–112]. Recognizing such vascular heterogeneity provides a mechanistic explanation for interindividual variability in blood pressure response, organ damage patterns, and residual cardiovascular risk despite adequate blood pressure control [5, 6, 107–112].

**Fig. 3:**
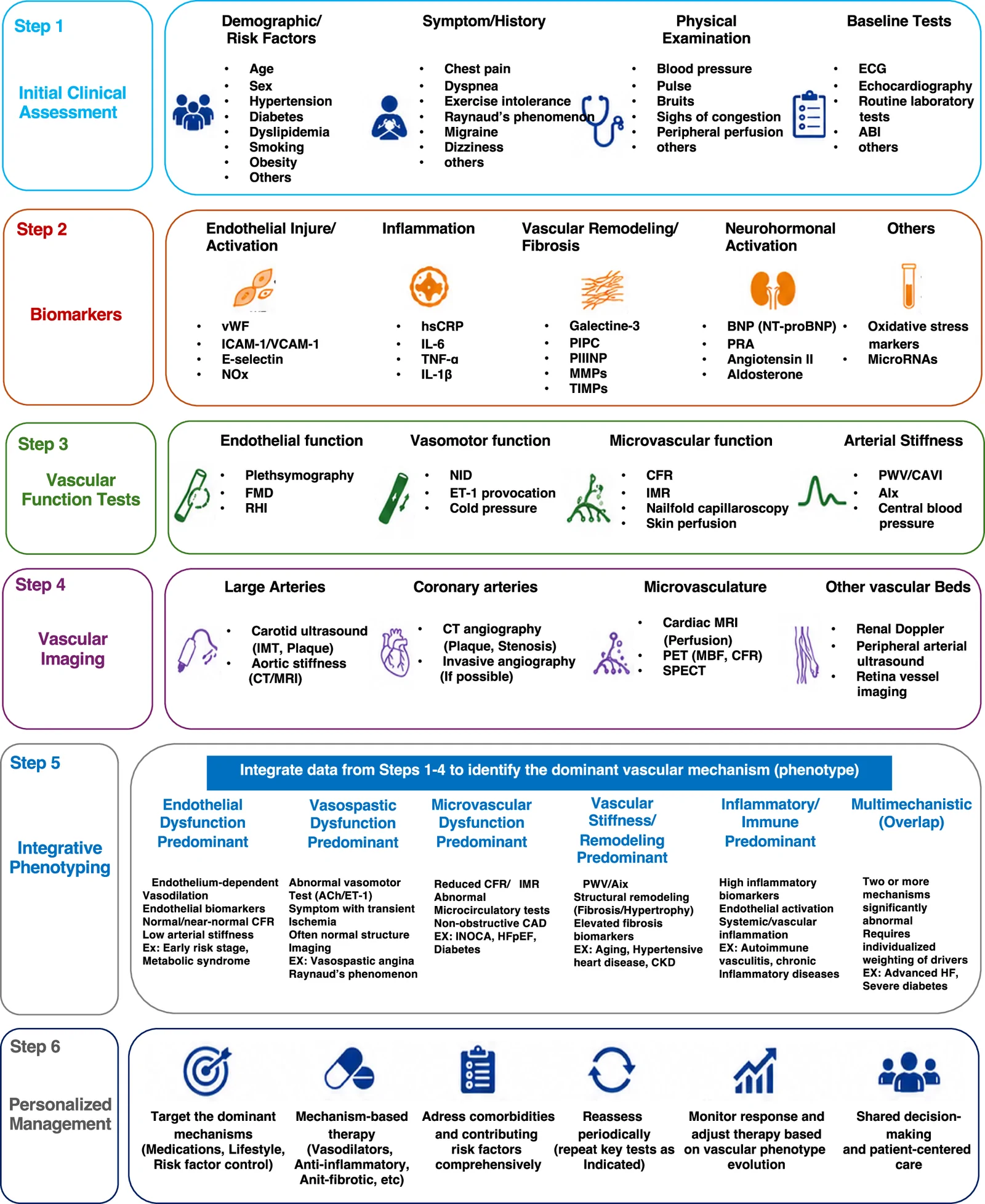
Proposed Clinical Algorithm for Vascular Phenotyping: Integrating Clinical Assessment, Vascular Function Tests, Biomarkers, and Imaging to Identify Dominant Vascular Mechanisms and Guide Personalized Management. ABI indicates ankle-brachial index, ACh acetylcholine, AIx augmentation index, CAD coronary artery disease, CAVI Cardio-ankle vascular index, CFR coronary flow reserve, CKD chronic kidney diseases, CPT cold pressure test, CT computed tomography, ECG electrocardiography, ET-1 endothelin-1, FMD flow-mediated vasodilation, HFpEF heart failure with preserved ejection fraction, hsCRP high-sensitivity C-reactive protein, ICAM-1 intercellular adhesion molecule-1, IL-6 interleukin-6, IMR index of microcirculatory resistance, IMT intima-media thickness, :INOCA ischemic non obstructive coronary artery disease, MBF myocardial blood flow, MMPs matrix metalloproteinases, MRI magnetic resonance imaging, NID nitroglycerine-induced vasodilation, NOx nitrite/nitrate, NT-pro BNP N-terminal pro brain natriuretic peptide, PET positron emission tomography, PIPC procollagen type C-terminal propeptide, PIIINP procollagen type III N-terminal propeptide, PRA plasma renin activity, PWV pulse wave velocity, RHI reactive hyperemia index, SPECT single photon emission computed tomography, TIMPs tissue inhibitor of metalloproteinases, TNF-α tumor necrosis factor-α, vWF von Willebrand factor, VCAM-1 vascular cell adhesion molecule-1

Figure 3 illustrates the proposed clinical algorithm for vascular phenotyping. The algorithm provides a conceptual framework that integrates routinely available clinical information, circulating biomarkers, vascular function testing, and multimodality vascular imaging to facilitate the identification of the predominant vascular phenotype in individual patients and support mechanism-based personalized management. The initial step involves a comprehensive clinical assessment, including blood pressure patterns, comorbidities, laboratory findings, and baseline cardiovascular risk profiles. The second step incorporates circulating biomarkers reflecting key pathophysiological processes, including endothelial dysfunction, inflammation, oxidative stress, vascular remodeling and fibrosis, and neurohormonal activation. The third step consists of non-invasive vascular function assessments, where available, including the evaluation of endothelial function, vasomotor function, microvascular function, and arterial stiffness to further refine vascular phenotypic characterization. The fourth step incorporates multimodal vascular imaging, including computed tomography, magnetic resonance imaging, positron emission tomography, single-photon emission computed tomography, and ultrasonography of the aorta, large arteries, cerebral and coronary arteries, renal arteries, peripheral arteries, and microvasculature. The final step synthesizes these multimodal data within a structured clinical framework that combines hemodynamic parameters, vascular functional measures, biomarkers, and indicators of target organ damage to identify the predominant vascular mechanism, such as pressure overload–driven large artery stiffness, endothelial dysfunction–predominant disease, or microvascular remodeling–dominant pathology. This integrated vascular phenotype may subsequently guide individualized therapeutic decision-making by aligning specific pathophysiological mechanisms with targeted pharmacological and lifestyle interventions, thereby extending clinical management beyond a uniform blood pressure–centered approach toward mechanism-based precision medicine. The accompanying text further discusses the strengths and limitations of this proposed framework and emphasizes that vascular phenotyping should always be interpreted within the context of the overall clinical presentation rather than relying on any single diagnostic modality. Importantly, the proposed algorithm is intended as a conceptual clinical framework rather than a validated diagnostic pathway. Prospective studies are therefore required to determine its clinical utility, reproducibility, and predictive value, as well as to establish whether phenotype-guided management can improve cardiovascular outcomes compared with conventional blood pressure–based treatment strategies.

Vascular phenotypes should not be regarded as discrete or mutually exclusive entities but rather as overlapping and dynamically interacting pathophysiological processes that frequently coexist within the same individual. Consequently, multiple vascular abnormalities—including arterial stiffness, endothelial dysfunction, and microvascular remodeling—may simultaneously contribute to cardiovascular risk, with their relative contributions varying according to disease stage, comorbidities, and the overall clinical context. This complexity is particularly evident in patients with chronic kidney disease or diabetes mellitus, in whom accelerated vascular aging, chronic inflammation, oxidative stress, and metabolic dysregulation collectively promote the concurrent development of both macrovascular and microvascular dysfunction. As a result, mixed vascular phenotypes are common in these populations, and the predominant pathophysiological mechanism may not be readily identifiable from a single cross-sectional assessment. Moreover, vascular phenotypes are inherently dynamic rather than static. Their characteristics may evolve over time in response to biological aging, progression of the underlying disease, cumulative exposure to cardiovascular risk factors, and therapeutic interventions. For example, vascular dysfunction initially characterized predominantly by impaired endothelial function may gradually progress toward increased arterial stiffness, structural vascular remodeling, and irreversible vascular damage. These considerations underscore the importance of longitudinal vascular phenotyping using repeated assessments rather than reliance on a single baseline evaluation. Serial characterization of vascular phenotypes may more accurately capture disease progression, monitor treatment-related changes in vascular biology, and facilitate mechanism-based adaptation of therapeutic strategies over time. Such a dynamic approach is expected to improve risk stratification and represents a key prerequisite for the future implementation of precision vascular medicine.

Recommended treatment strategies for each vascular phenotype are of considerable clinical significance. Table 1 provides a structured summary of the major vascular phenotypes, their predominant pathophysiological mechanisms, representative diagnostic features, potential therapeutic approaches, and the current level of supporting clinical evidence. The table also distinguishes interventions supported by randomized clinical trials and current guideline recommendations from those based primarily on mechanistic studies, observational evidence, or emerging clinical data, thereby clarifying the strength of evidence for each therapeutic strategy. The accompanying text further discusses the mechanistic rationale underlying phenotype-guided therapy. For example, renin–angiotensin–aldosterone system inhibitors may improve endothelial dysfunction and attenuate vascular remodeling, calcium channel blockers may preferentially reduce functional arterial stiffness, and metabolic or anti-inflammatory interventions may provide additional benefits in selected vascular phenotypes. Overall, the strength of evidence varies considerably across phenotypes and therapeutic classes, ranging from robust cardiovascular outcome data for established antihypertensive therapies to limited evidence supporting phenotype-specific vascular targeting. Despite the strong biological rationale and the growing body of supporting evidence, several important knowledge gaps remain. These include the lack of standardized definitions for vascular phenotypes, limited validation of diagnostic thresholds, and the absence of randomized controlled trials directly evaluating phenotype-guided treatment algorithms. Consequently, future prospective, mechanism-oriented clinical trials are needed to determine whether vascular phenotype–based treatment selection can improve cardiovascular outcomes beyond those achieved with conventional blood pressure–based management. Ultimately, in patients with hypertension, therapeutic decisions may evolve toward targeting the predominant vascular mechanism rather than relying solely on blood pressure reduction.

**Table 1:** Major vascular phenotypes, their predominant pathophysiological mechanisms, representative diagnostic features, potential therapeutic approaches, and the current level of supporting clinical evidence

Phenotype	Main mechanism	Preferred therapy	Evidence
Endothelial dysfunction	Decrease in NO bioavailability	RAAS inhibitors, Statins, SGLT2 inhibitors, Lifestyle modification	Strong
Vasospastic dysfunction	Hyperreactive VSMC	Calcium channel blockers, nitrates	Strong
Microvascular dysfunction	Impaired microcirculation	RAAS inhibitors, SGLT2 inhibitors, GLP-1 RAs,	Moderate
Arterial stiffness	Fibrosis, Calcification	RAAS inhibitors, Lifestyle modification	Moderate
Inflammatory/ Immune	Chronic inflammation Impaired immune system	Anti-inflammatory therapy, Colchicine, IL-1 inhibitor	Emerging

*NO* indicates nitric oxide, *RAAS* renin-angiotensin-aldosterone system, *SGLT2* sodium–glucose cotransporter 2, *VSMC* vascular smooth muscle cell, *GLP1 RAs* glucagon-like peptide-1 receptor agonists, *IL-1* interleukin-1

Recent advances suggest that hypertension may not be a uniform disease entity but rather a spectrum of vascular pathophysiological states. In this context, assessment of vascular characteristics such as arterial stiffness, endothelial function, and microvascular remodeling may provide additional information beyond conventional blood pressure measurements. Such phenotypic stratification has the potential to guide more individualized therapeutic strategies, for example by identifying patients who may preferentially benefit from RAAS inhibition, calcium channel blockade, or interventions targeting vascular stiffness and endothelial dysfunction. However, at present, vascular phenotyping should be considered an adjunct to, rather than a replacement for, established guideline-directed blood pressure management. Further prospective studies are needed to determine whether phenotype-guided treatment strategies can improve cardiovascular outcomes compared with standard care.

### Endothelial-dominant phenotype

The endothelial-dominant phenotype is characterized primarily by impaired endothelium-dependent vasodilation in the absence of advanced structural arterial remodeling [9–12, 69–72, 82–87]. This phenotype is frequently observed in early-stage hypertension, metabolic syndrome, obesity, insulin resistance, and in individuals with high-normal blood pressure who already exhibit elevated cardiovascular risk [9–12, 82–87]. At the molecular level, reduced NO bioavailability is central [13–16, 73, 74]. Impaired eNOS phosphorylation, increased oxidative inactivation of NO, accumulation of endogenous eNOS inhibitors such as asymmetric dimethylarginine, and oxidation of BH_4_ collectively limit NO production [16, 73–76]. In parallel, activation of ENaC in high-sodium environments increases endothelial cortical stiffness, blunting shear stress–mediated NO release [53, 54]. Degradation of the endothelial glycocalyx further disrupts mechanotransduction and sodium buffering capacity, reinforcing endothelial vulnerability [77–81]. Functionally, patients with this phenotype demonstrate impaired FMD while PWV and central blood pressure may remain within near-normal ranges [25–35, 82–87]. The hemodynamic abnormality is therefore primarily functional rather than structural. However, sustained endothelial dysfunction increases basal vascular tone, enhances responsiveness to vasoconstrictors such as Ang II and ET-1, and promotes low-grade inflammation [15, 16, 73, 74]. Over time, these abnormalities may transition into structural vascular remodeling and sustained hypertension [25–39]. Clinically, this phenotype may represent a reversible stage of vascular dysfunction. Therapeutic strategies that restore NO signaling—such as RAAS blockade, exercise training, weight reduction, and dietary salt moderation—may be particularly effective in this group [107, 113–122]. Failure to identify and treat endothelial dysfunction early may permit progression toward more advanced structural phenotypes [108–112].

### Stiffness-dominant phenotype

The stiffness-dominant phenotype is defined by increased arterial stiffness and abnormal central hemodynamics that are often disproportionate to brachial blood pressure values [25–39]. This phenotype is commonly observed in aging, chronic kidney disease, diabetes, and long-standing hypertension [29, 37, 108–112]. Structurally, this phenotype reflects extracellular matrix remodeling within large conduit arteries. Increased collagen deposition, elastin fragmentation, and advanced glycation end-product crosslinking reduce arterial compliance [36, 37]. These processes are driven by chronic oxidative stress, activation of transforming growth factor-β signaling, and low-grade inflammation [40–49, 75, 88–100]. In addition to structural remodeling, functional stiffness contributes significantly. Enhanced vascular smooth muscle tone mediated by rhoA/rho-kinase signaling and calcium sensitization increases wall tension independently of fixed fibrosis [37–39]. Hemodynamically, increased PWV accelerates wave reflection, augmenting central systolic blood pressure and pulse pressure [25–35]. Although brachial blood pressure may appear controlled, central aortic pressure may remain elevated, exposing the left ventricle and microvascular beds to excessive pulsatile stress [29–33]. This mechanism explains the strong association between arterial stiffness and cerebrovascular injury, cognitive decline, and chronic kidney disease progression [27–33]. In this phenotype, hypertension is maintained not only by peripheral resistance but also by abnormal pulsatile load. Therapeutic approaches that reduce arterial stiffness—particularly RAAS inhibition and MR antagonism—may offer benefits beyond simple brachial blood pressure reduction [107, 113]. However, advanced structural remodeling may limit reversibility, emphasizing the importance of early intervention [108–112].

### Microvascular-dominant phenotype

The microvascular-dominant phenotype is characterized by structural and functional alterations in resistance vessels and capillary networks that increase systemic vascular resistance [19–24, 101–104]. Microvascular rarefaction, defined as a reduction in capillary density, represents a hallmark of this phenotype [19–24]. Both structural rarefaction, involving actual capillary loss, and functional rarefaction, characterized by impaired capillary recruitment, contribute to elevated peripheral resistance [19–24, 101, 102]. Oxidative stress, endothelial apoptosis, reduced vascular endothelial growth factor signaling, and chronic vasoconstrictor predominance drive capillary dropout [19–24, 75, 88–104]. Increased ET-1 activity and enhanced precapillary arteriolar tone limit perfusion even in structurally intact networks [19–24, 101–104]. As parallel microvascular channels are lost, total peripheral resistance increases, amplifying blood pressure responses to physiological stress [19–24]. Importantly, microvascular dysfunction contributes directly to target organ damage. In the kidney, capillary rarefaction accelerates glomerulosclerosis; in the brain, it promotes white matter injury and small vessel disease; in the heart, it impairs coronary microvascular reserve [101–104]. Thus, this phenotype links systemic hypertension with organ-specific microcirculatory injury. Patients with this phenotype may demonstrate relatively preserved large artery stiffness in early stages but exhibit impaired reactive hyperemia and elevated systemic vascular resistance [60–63]. Interventions that stimulate angiogenesis, improve metabolic function, and enhance microvascular perfusion—such as exercise and certain metabolic therapies—may be particularly beneficial [115–121].

### Inflammatory phenotype

The inflammatory phenotype reflects a dominant role of immune activation in driving vascular dysfunction and blood pressure elevation [40–49]. This phenotype is frequently associated with salt sensitivity, obesity, and metabolic dysregulation [42–49]. High-salt environments promote serum- and glucocorticoid-regulated kinase 1 activation within T cells, enhancing differentiation toward the Th17 lineage [42, 43]. IL-17 impairs endothelial NO production, increases oxidative stress via NADPH oxidase activation, and upregulates vascular adhesion molecules that facilitate leukocyte infiltration [44, 45]. Concurrently, local MR activation within endothelial and smooth muscle cells promotes oxidative stress, fibrosis, and inflammatory gene transcription [50–57]. In this phenotype, hypertension arises in part from immune-mediated vascular injury rather than purely from neurohumoral or renal mechanisms [40–49]. Elevated inflammatory biomarkers and heightened oxidative stress are frequently observed [40–49, 75, 76, 88–100]. Even when systemic RAAS activity is suppressed by high salt intake, local tissue signaling may remain active, perpetuating vascular inflammation [50–57]. Clinically, patients with inflammatory-dominant hypertension may respond particularly well to salt restriction and MR antagonism [50–57]. Persistent immune activation may explain residual vascular dysfunction despite adequate blood pressure control [5, 6, 108–112]. These vascular phenotypes should not be interpreted as discrete categories but as dominant mechanistic axes within a continuum of vascular pathology [7, 8]. Early hypertension may be primarily endothelial-dominant, progressing toward stiffness- and microvascular-dominant states with aging and chronic exposure to hemodynamic stress [108–112]. Immune activation may intersect with each phenotype, amplifying redox imbalance and structural remodeling [40–57]. A vessel-centric classification framework thus provides a biologically coherent explanation for therapeutic heterogeneity and residual cardiovascular risk [5, 6, 107]. By identifying the dominant vascular mechanism in an individual patient, clinicians may move beyond uniform blood pressure targets toward mechanism-guided hypertension management [107–112].

## Clinical assessment of vascular function

**Fig. 4:**
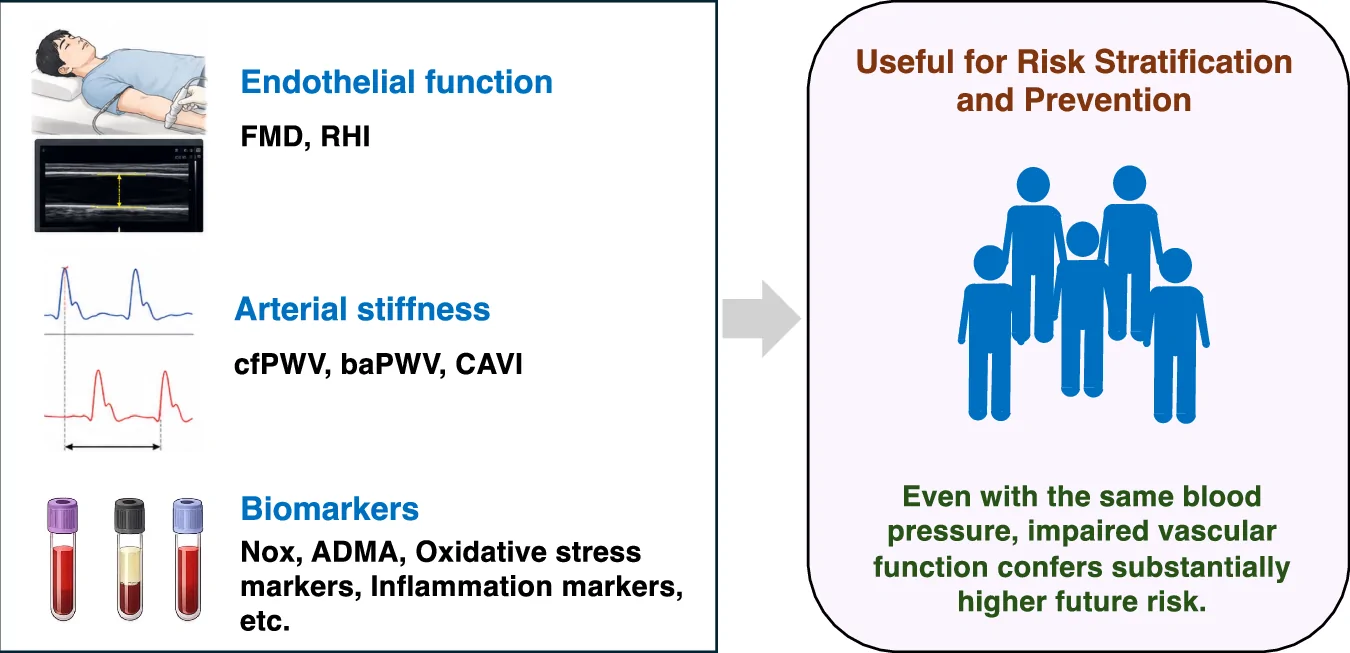
Assessment of Vascular Function: from Bench to Bedside. FMD indicates flow-mediated vasodilation, RHI reactive hyperemia index, cFPWV carotid-femoral pulse wave velocity, baPWV brachial-ankle PWV, CAVI cardio ankle vascular index, Nox nitrite/nitrate, ADMA asymmeteric dimethylarginen

The transition from a pressure-centric to a vessel-centric paradigm in hypertension requires reliable and clinically actionable methods for assessing vascular function (Fig. 4) [58–68]. A variety of macrovascular and microvascular techniques, along with circulating biomarkers, have been developed to characterize different dimensions of vascular health [9–12, 19–39, 69–72, 82–87, 101–104]. However, despite robust mechanistic rationale and prognostic evidence, integration of these measures into routine clinical practice remains limited [4, 58–68]. A comprehensive understanding of their physiological basis, strengths, and limitations is essential for advancing precision vascular medicine [107–112].

### Macrovascular methods

Macrovascular assessment primarily evaluates endothelial function and arterial stiffness in conduit arteries (Fig. 4) [58–68]. Among these techniques, FMD remains the most extensively studied noninvasive method of assessing endothelium-dependent vasodilation [9–12, 58, 64, 69–72, 82–87]. FMD measures brachial artery dilation in response to reactive hyperemia, reflecting shear stress–induced NO release [13–16, 73, 74, 85, 86]. Impaired FMD has been shown to predict incident hypertension and cardiovascular events, supporting its role as a functional marker of early vascular injury [82–87]. Importantly, FMD captures dynamic endothelial responsiveness rather than structural remodeling, making it particularly informative in early or endothelial-dominant phenotypes [82–87]. Nitroglycerine-mediated dilation (NID) complements FMD by assessing endothelium-independent vasodilation through direct smooth muscle relaxation [58, 63]. Comparison between FMD and NID allows differentiation between endothelial dysfunction and smooth muscle impairment, providing mechanistic insight into the site of vascular abnormality [9, 58–64, 82]. Arterial stiffness is commonly assessed by PWV, which quantifies the speed of pulse wave propagation along the arterial tree [25–35]. Carotid–femoral PWV is widely regarded as the gold standard for evaluating aortic stiffness and has strong prognostic value for cardiovascular events [25–28]. Increased PWV reflects both structural remodeling, such as collagen accumulation and elastin degradation, and functional smooth muscle tone [36–39]. CAVI offers an alternative stiffness parameter that is less dependent on instantaneous blood pressure at the time of measurement, potentially providing a more stable index of intrinsic arterial stiffness [34]. Central blood pressure measurement, derived from applanation tonometry or oscillometric devices with transfer function algorithms, provides insight into aortic systolic pressure and pulse pressure amplification [29–33]. Central blood pressure may better reflect left ventricular afterload and cerebrovascular pulsatile stress than brachial blood pressure, particularly in stiffness-dominant phenotypes [29–33]. Together, these macrovascular methods enable characterization of conduit artery function and central hemodynamics, offering mechanistic information beyond peripheral blood pressure values.

### Microvascular evaluation

Microvascular assessment focuses on resistance vessels and capillary networks that directly determine systemic vascular resistance and tissue perfusion (Fig. 4) [19–24, 101–104]. Because microvascular dysfunction may precede large artery remodeling, these techniques are particularly relevant in early or microvascular-dominant phenotypes [19–24, 101–104]. The reactive hyperemia index (RHI), obtained using peripheral arterial tonometry, evaluates digital pulse volume changes following transient ischemia [60–62]. RHI reflects microvascular endothelial function and NO–mediated vasodilation in resistance vessels [60–62]. Although less operator-dependent than FMD, it may capture distinct aspects of microvascular reactivity rather than conduit artery function [60–62]. Retinal vascular imaging provides a noninvasive window into the microcirculation. Quantitative assessment of retinal arteriolar narrowing, arteriovenous ratio, and microvascular remodeling correlates with systemic hypertension and predicts cerebrovascular events [101–104]. Because retinal vessels share embryological and anatomical similarities with cerebral microvessels, retinal imaging offers insight into microvascular damage in target organs [101–104]. Skin microcirculatory assessment, including laser Doppler flowmetry and capillaroscopy, enables evaluation of capillary density and recruitment [20–24]. Reduced capillary density reflects structural rarefaction, whereas impaired recruitment indicates functional dysregulation [19–24]. These measures provide direct evidence of microvascular remodeling, which may contribute to elevated peripheral resistance and organ injury [19–24, 101–104]. Microvascular evaluation thus complements macrovascular assessment by capturing resistance vessel pathology that may not be evident in large artery measurements [19–24, 60–62, 101–104].

### Biomarkers

Circulating biomarkers offer an accessible means of assessing vascular injury and may facilitate large-scale phenotyping (Fig. 4) [65–68]. Endothelial microparticles, released during endothelial activation or apoptosis, reflect endothelial damage and have been associated with hypertension severity and cardiovascular risk [65–68]. Markers of glycocalyx degradation, such as circulating syndecan-1 and heparan sulfate fragments, provide insight into endothelial surface integrity [77–81]. Because glycocalyx disruption precedes overt structural remodeling, these markers may identify early vascular injury [77–81]. Oxidative stress biomarkers, including urinary 8-hydroxy-2′-deoxyguanosine and circulating oxidized lipoproteins, indicate redox imbalance within the vascular wall [75, 76, 88–100]. Inflammatory cytokines, such as IL-6 and IL-17, reflect immune activation and may characterize inflammatory-dominant phenotypes [40–49]. While individual biomarkers lack sufficient specificity to define vascular phenotypes independently, integrated panels combining functional and molecular markers may enhance precision risk stratification [65–68]. The future development of multimodal biomarker algorithms may enable noninvasive vascular endotyping.

### Practical barriers and implementation

Despite compelling mechanistic and prognostic evidence, several barriers limit the widespread adoption of vascular function assessment in clinical practice [58–68]. Standardization remains a major challenge, particularly for FMD, which is sensitive to operator technique, environmental conditions, and patient preparation [58–68, 85, 86]. Inter-device variability and methodological heterogeneity complicate cross-study comparisons [58–68]. Cost, time requirements, and limited reimbursement further restrict routine use. Moreover, current hypertension guidelines primarily focus on brachial blood pressure thresholds and global cardiovascular risk estimation, with minimal integration of vascular function metrics as therapeutic decision tools [1–4]. To transition toward vessel-centric hypertension care, future studies must establish standardized protocols, demonstrate reproducibility across centers, and define actionable thresholds linked to clinical outcomes [58–68]. Incorporation of vascular endpoints into interventional trials will be essential to determine whether improving vascular function independently reduces cardiovascular events [107]. Ultimately, the clinical implementation of vascular assessment requires a shift from viewing these measures as research tools to recognizing them as integral components of hypertension phenotyping and precision management [107–112].

## Therapeutic targeting of vascular biology

**Fig. 5:**
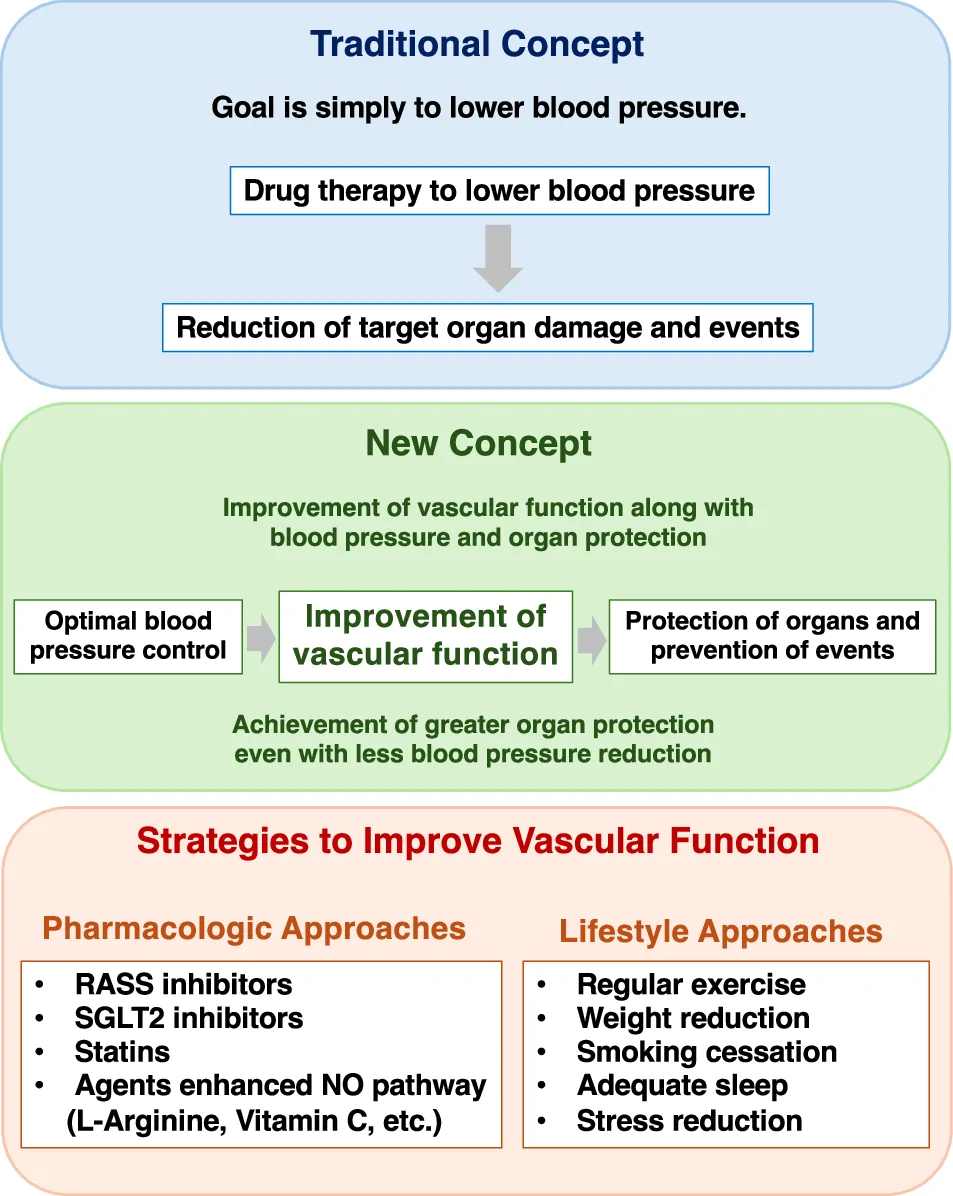
Paradigm Shift in Hypertension Treatment: Add Blood Pressure Control, Prioritize Vascular Function Improvement. RAAS indicates renin-angiotensin-aldosterone system; SGLT2 sodium–glucose cotransporter 2, NO nitric oxide

The recognition that hypertension is fundamentally a disorder of the vascular wall has profound therapeutic implications (Fig. 5) [7–9, 107]. Although antihypertensive drugs are traditionally evaluated according to their capacity to lower brachial blood pressure, many pharmacologic and non-pharmacologic interventions exert direct effects on endothelial function, oxidative stress, inflammation, and arterial stiffness that extend beyond simple hemodynamic reduction [107, 113–122]. Importantly, improvement in vascular biology does not always parallel the magnitude of blood pressure reduction [5, 6, 107]. Thus, a vessel-centric therapeutic framework requires evaluation of both pressure lowering and vascular restoration.

### Pharmacologic interventions

RAAS inhibitors remain central to hypertension management, but their benefits extend beyond systemic vasodilation and natriuresis [1–4]. Ang II stimulates NADPH oxidase activity, promotes eNOS uncoupling, enhances vascular smooth muscle proliferation, and activates inflammatory signaling pathways [40–49, 75, 76, 88–100]. By attenuating Ang II–mediated oxidative stress, angiotensin-converting enzyme inhibitors and angiotensin receptor blockers restore NO bioavailability, reduce endothelial activation, and improve FMD independent of their blood pressure effects [9, 107, 113]. Long-term RAAS inhibition also mitigates extracellular matrix remodeling and collagen deposition, contributing to reductions in arterial stiffness [25–39, 107, 113]. MR antagonists further illustrate the dissociation between blood pressure lowering and vascular repair [50–57]. Aldosterone promotes oxidative stress, fibrosis, and inflammatory gene expression within endothelial and smooth muscle cells [50–52]. MR blockade reduces NADPH oxidase expression, suppresses collagen synthesis, and improves endothelial function even in settings where systemic RAAS activity is not overtly elevated [50–57]. In salt-sensitive and inflammatory-dominant phenotypes, vascular benefits of MR antagonism may be disproportionate to changes in brachial blood pressure [50–57]. Sodium–glucose cotransporter 2 (SGLT2) inhibitors, initially developed as glucose-lowering agents, have demonstrated robust cardiovascular protection [115–117]. Although modest reductions in blood pressure contribute to their clinical benefits, emerging data suggest that improvements in vascular function may play an important role [115–117]. SGLT2 inhibition reduces oxidative stress, enhances endothelial NO signaling, improves arterial stiffness, and modulates inflammatory pathways [115–117]. Improvement in central hemodynamics and PWV has been observed even when brachial blood pressure reductions are modest, suggesting a direct vascular effect [115–117]. Glucagon-like peptide-1 (GLP-1) receptor agonists similarly exhibit pleiotropic vascular actions [118]. Beyond metabolic improvements and weight reduction, GLP-1 receptor activation enhances endothelial NO production, attenuates oxidative stress, and reduces vascular inflammation [118]. In experimental models, GLP-1 signaling suppresses adhesion molecule expression and improves endothelial-dependent vasodilation [118]. These effects may contribute to observed reductions in major cardiovascular events, particularly in patients with metabolic and inflammatory phenotypes [118]. Collectively, these pharmacologic agents demonstrate that targeting vascular biology can complement traditional pressure reduction. The degree to which vascular restoration mediates long-term cardiovascular benefit remains an important area for investigation [107–112].

### Lifestyle and salt modulation

Lifestyle modification represents a foundational therapy in hypertension, yet its vascular mechanisms are often underappreciated [119–121]. Regular aerobic exercise enhances shear stress–mediated NO production, upregulates eNOS expression, improves mitochondrial function, and reduces oxidative stress [85, 86, 119–121]. Exercise also stimulates angiogenesis through vascular endothelial growth factor signaling, potentially reversing microvascular rarefaction [19–24, 101–104]. Improvements in endothelial function and arterial stiffness frequently precede significant reductions in resting blood pressure, underscoring a direct vascular effect [119–121]. Weight reduction improves endothelial function through decreased adipose-derived inflammatory cytokines, reduced oxidative stress, and improved insulin sensitivity [40–49, 119–121]. Adiposity is associated with chronic low-grade inflammation and endothelial activation; weight loss attenuates these processes and improves NO bioavailability [119–121]. Dietary salt restriction exerts vascular effects that extend beyond volume reduction [42–57]. High sodium intake increases endothelial stiffness via ENaC activation, disrupts the glycocalyx, and promotes Th17-mediated immune activation [42–57, 77]. Reduction in salt intake restores endothelial shear responsiveness, reduces oxidative stress, and attenuates inflammatory signaling within the vascular wall [42–57, 77]. In salt-sensitive individuals, improvements in endothelial function may precede or exceed changes in blood pressure [53–57, 77]. Thus, lifestyle interventions may restore vascular homeostasis through mechanisms that are partially independent of brachial blood pressure reduction, highlighting their importance within a vessel-centric therapeutic model [119–121].

### Emerging vascular-directed therapies

Advances in understanding redox and immune mechanisms have stimulated development of therapies specifically targeting vascular pathology [40–57, 75, 76, 88–100]. Novel anti-inflammatory strategies aimed at modulating interleukin signaling, immune cell activation, or inflammasome pathways may offer benefit in inflammatory-dominant hypertension [40–49]. Redox-modulating therapies targeting NADPH oxidase activity, mitochondrial oxidative stress, or nuclear factor erythroid 2–related factor 2 signaling represent additional promising avenues [75, 76, 88–100]. Endothelial-targeted strategies aimed at restoring NO signaling, stabilizing BH_4_ levels, or protecting the glycocalyx are under investigation [74–81]. Agents that inhibit rho-kinase signaling may reduce functional arterial stiffness and improve vascular compliance independently of structural remodeling [37–39]. Importantly, the evaluation of these therapies should not rely solely on brachial blood pressure reduction as the primary endpoint. Incorporation of vascular function measures—such as FMD, PWV, central blood pressure, and microvascular perfusion—may provide mechanistic insight and identify early therapeutic effects [25–39, 58–68]. A key principle emerging from a vessel-centric framework is that blood pressure reduction and vascular restoration are not always concordant [5, 6, 107]. Some agents produce substantial reductions in brachial blood pressure with minimal improvement in endothelial function or arterial stiffness, whereas others exert significant vascular benefits despite modest blood pressure changes [9, 107, 113–122]. Residual cardiovascular risk in treated hypertension may reflect incomplete repair of vascular dysfunction rather than inadequate pressure control [5, 6, 108–112]. Therefore, future hypertension management should incorporate dual evaluation: the magnitude of blood pressure reduction and the degree of vascular function improvement [9, 107]. Aligning therapeutic strategies with dominant vascular phenotypes may enhance precision, reduce residual risk, and redefine treatment success beyond numerical blood pressure targets [107–112].

## Residual risk and the concept of vascular age

Despite substantial advances in antihypertensive therapy and widespread achievement of guideline-recommended blood pressure targets, a considerable proportion of patients with well-controlled brachial blood pressure continue to experience cardiovascular events [5, 6]. This persistent residual risk challenges the assumption that normalization of brachial blood pressure equates to restoration of vascular health [5, 6, 108–112]. Increasing evidence suggests that structural and functional abnormalities within the vascular wall may persist despite apparent hemodynamic control [107–112]. Endothelial dysfunction frequently remains detectable in patients whose office and ambulatory blood pressure values are within recommended ranges [9–12, 69–72, 82–87]. Reduced NO bioavailability, impaired shear stress–mediated vasodilation, and low-grade vascular inflammation may continue even after successful pharmacologic blood pressure lowering [13–16, 40–49, 73–76, 88–100]. Similarly, arterial stiffness, once established through collagen accumulation and elastin fragmentation, may only partially regress despite normalization of peripheral pressure [25–39]. Because arterial stiffness amplifies pulsatile load and central systolic pressure, patients may remain exposed to adverse central hemodynamics even when brachial blood pressure appears controlled [29–33]. These observations have led to the emerging concept of “vascular age”, which reflects the biological condition of the arterial system rather than chronological age alone [108–112]. Vascular age integrates both structural indices—such as PWV, central pulse pressure, and arterial remodeling—and functional indices, including endothelial-dependent vasodilation and microvascular reactivity [25–39, 58–68]. An individual with well-controlled blood pressure but impaired FMD and elevated PWV may exhibit a vascular age substantially older than their chronological age. The divergence between chronological age and vascular age may help explain heterogeneity in cardiovascular outcomes among patients with similar blood pressure profiles [5, 6, 108–112]. For example, two individuals with identical brachial systolic blood pressure may differ markedly in central aortic pressure, arterial stiffness, and endothelial function, leading to different risks of stroke, heart failure, or renal decline [25–39]. In this context, blood pressure becomes only one dimension of a broader vascular risk phenotype [107]. The concept of masked vascular dysfunction further expands this framework. Some individuals, particularly those with metabolic abnormalities or inflammatory activation, may display normal or near-normal blood pressure but exhibit significant endothelial impairment or microvascular rarefaction [19–24, 40–49, 101–104]. Such patients may represent a “preclinical vascular hypertension” state, in which vascular injury precedes sustained pressure elevation [7–9, 107]. Failure to recognize this stage may delay preventive intervention until structural remodeling becomes less reversible [108–112]. Incorporating vascular health metrics into cardiovascular risk assessment may therefore refine prevention strategies [58–68]. Measurement of arterial stiffness, central hemodynamics, and endothelial function could identify individuals whose vascular biology remains abnormal despite adequate blood pressure control [25–39, 58–68]. In these patients, therapeutic intensification aimed at vascular restoration—rather than further blood pressure lowering alone—may be warranted [113–122]. From a longitudinal perspective, vascular age may also serve as a dynamic marker of treatment success. Whereas chronological age increases inexorably, vascular age may stabilize or even regress with effective therapy targeting oxidative stress, inflammation, and structural remodeling [40–57, 75, 76, 88–100]. Demonstrating improvement in vascular age metrics may provide a more comprehensive indicator of cardiovascular protection than blood pressure reduction alone [107–112]. Ultimately, redefining treatment success in hypertension may require a shift from achieving numerical blood pressure targets to achieving vascular health restoration [9, 107]. Integrating vascular age into clinical algorithms could enable earlier intervention, more individualized therapy, and improved prediction of long-term outcomes [108–112]. Recognizing that well-controlled blood pressure does not necessarily imply vascular normalization is a critical step toward a truly vessel-centric approach to hypertension management [7–9, 107].

## Future perspectives

### Precision vascular medicine

**Fig. 6:**
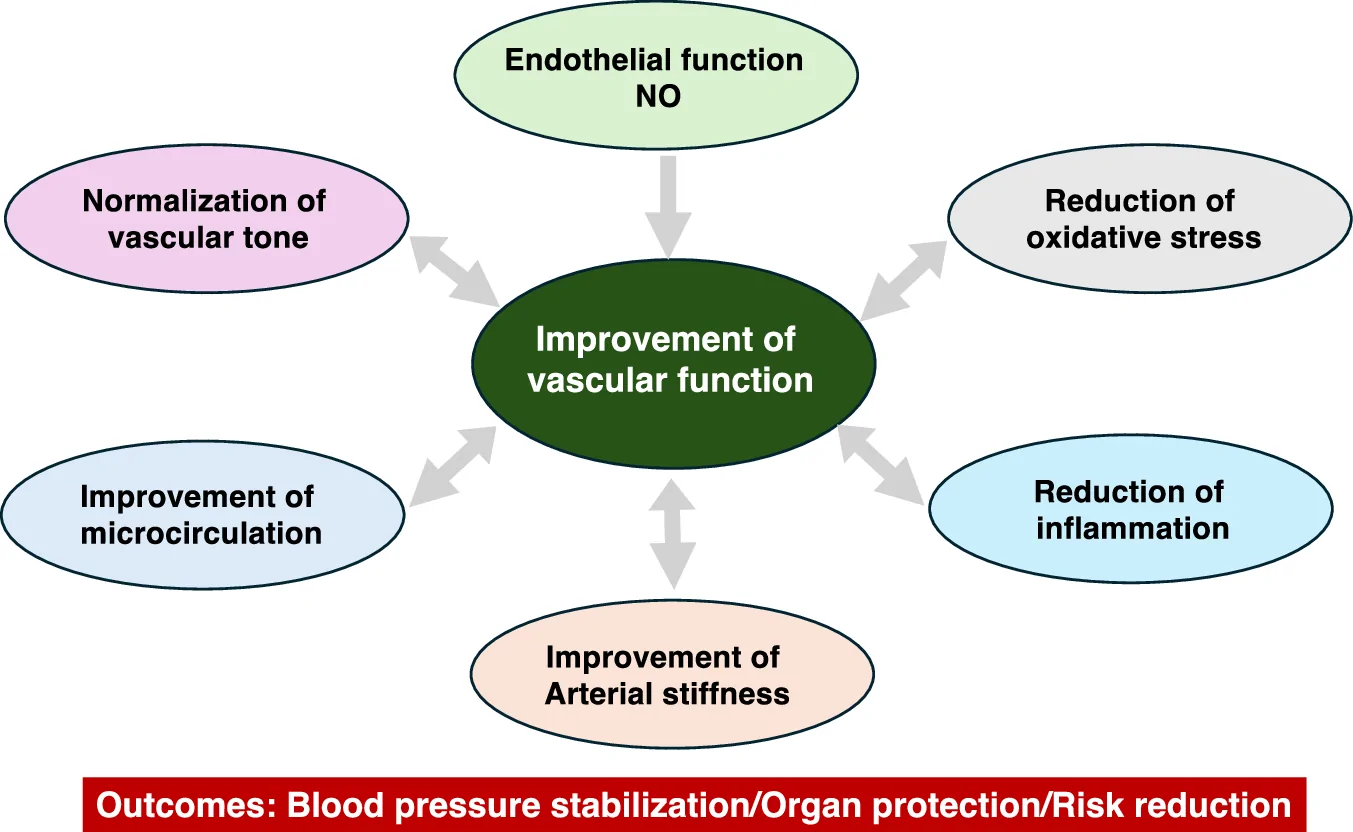
Improvement of vascular function

The growing recognition of vascular heterogeneity in hypertension opens the possibility of precision vascular medicine [7–9, 107]. Rather than treating hypertension as a uniform elevation of blood pressure requiring standardized pharmacologic escalation, a vessel-centric framework suggests that therapy should be guided by the dominant vascular phenotype [107–112]. Patients exhibiting endothelial-dominant dysfunction may benefit most from interventions that restore NO bioavailability and reduce oxidative stress, whereas those with stiffness-dominant phenotypes may require therapies targeting extracellular matrix remodeling and central hemodynamics [13–16, 25–39, 73, 74]. Similarly, individuals with inflammatory-dominant profiles may respond preferentially to MR antagonism or salt restriction strategies, while microvascular-dominant patients may require interventions that enhance angiogenesis and improve resistance vessel reactivity [19–24, 40–57, 101–104]. Such stratification moves beyond conventional risk factor counting and toward mechanistic endotyping [107]. Importantly, vascular phenotypes are dynamic and may evolve over time; thus, precision vascular medicine would require periodic reassessment of vascular function [58–68]. This approach aligns with the broader movement toward personalized cardiovascular therapy but places vascular biology at its core [107–112]. In this paradigm, treatment success would be defined not only by blood pressure normalization but also by restoration of endothelial function, reduction of arterial stiffness, and improvement of microvascular integrity (Fig. 6) [9–12, 19–39, 69–72, 82–87, 101–104]. The integration of vascular phenotyping into clinical decision-making represents a fundamental reorientation of hypertension management [107].

### Artificial intelligence (AI) integration

AI has the potential to transform vascular assessment and risk prediction in hypertension. AI-assisted imaging analysis can enhance reproducibility and reduce operator dependency in techniques such as FMD, PWV assessment, and retinal microvascular imaging [58–68]. Automated edge-detection algorithms and machine learning–based waveform analysis may standardize measurements across centers, addressing one of the major barriers to widespread implementation [58–68]. Beyond imaging, AI-driven biomarker analytics can integrate multidimensional data—including vascular function indices, inflammatory markers, metabolic parameters, and genetic information—to generate individualized vascular risk profiles [40–49, 65–68]. Machine learning models may identify latent vascular phenotypes not readily apparent through conventional classification [107]. Longitudinal AI systems could track changes in vascular age over time, detecting subclinical deterioration before overt blood pressure elevation occurs [108–112]. In this context, hypertension management may evolve toward continuous vascular monitoring rather than episodic blood pressure measurement alone [107]. Importantly, AI integration should not replace mechanistic understanding but rather enhance it by uncovering complex, nonlinear interactions among vascular, metabolic, and immune pathways [40–57, 75, 76, 88–100].

### Trial design innovation

The incorporation of vascular function endpoints into clinical trials represents a critical step toward accelerating translational progress [9, 107]. Traditionally, antihypertensive trials have prioritized brachial blood pressure reduction as the primary efficacy metric [1–6]. However, this approach may overlook mechanistic benefits that precede or exceed changes in peripheral blood pressure [113–122]. Including measures such as FMD, PWV, central aortic pressure, or microvascular perfusion as predefined endpoints could clarify whether a therapy exerts direct vascular repair effects [25–39, 58–68]. Early-phase trials incorporating vascular endpoints may enable rapid mechanistic evaluation and more efficient selection of promising interventions [107]. Moreover, phenotype-guided trial design may reduce heterogeneity and enhance statistical power. For example, enrolling patients with inflammatory-dominant hypertension into trials of immune-modulating therapies may increase the likelihood of detecting clinically meaningful effects [40–49]. Similarly, central hemodynamic endpoints may be particularly relevant in stiffness-dominant populations [25–39]. Redefining surrogate endpoints to include vascular restoration—rather than relying exclusively on blood pressure reduction—may shift the paradigm of antihypertensive drug development and evaluation [107–112].

### Redefining hypertension criteria

Perhaps the most provocative implication of a vessel-centric framework is the potential redefinition of hypertension itself [7, 8]. Current diagnostic criteria rely exclusively on blood pressure thresholds measured at the brachial artery [1–4]. While practical and epidemiologically validated, this definition does not capture early vascular dysfunction that may precede sustained pressure elevation [7–9, 107]. Future definitions of hypertension may incorporate measures of vascular dysfunction—such as impaired endothelial-dependent vasodilation, elevated arterial stiffness, or evidence of microvascular rarefaction—alongside blood pressure thresholds [9–12, 19–39, 69–72, 82–87, 101–104]. Individuals with normal or high-normal blood pressure but significant vascular impairment could be recognized as having “preclinical vascular hypertension” or a stage of vascular dysfunction that warrants early intervention [107–112]. Such a redefinition would not eliminate blood pressure measurement as a central diagnostic tool but would expand the conceptual framework to include vascular biology [7, 8]. This shift may enable earlier identification of at-risk individuals and promote preventive strategies before irreversible structural remodeling occurs [108–112]. While the implementation of such criteria would require robust validation and cost-effectiveness analyses, the integration of vascular function into diagnostic algorithms represents a logical extension of accumulating mechanistic evidence [9, 107]. This future-oriented perspective underscores a central theme of this review: hypertension should not be viewed solely as a disorder of elevated pressure, but as a complex vascular disease requiring multidimensional assessment and intervention [7, 8]. Precision vascular medicine, supported by AI integration and innovative trial design, may redefine both diagnosis and therapeutic success in the coming decade [107–112].

### Standardization and clinical implementation of vascular function assessment

The broader implementation of vascular function assessment, particularly FMD, in both research and clinical practice requires rigorous standardization of measurement methodology. Establishing standardized and reproducible imaging and acquisition protocols is essential to minimize inter- and intra-observer variability and to improve the comparability of measurements across institutions. Equally important is the development of formal operator training and certification programs to ensure methodological consistency and maintain high-quality data acquisition. The adoption of automated or semi-automated image analysis platforms represents another important step toward reducing observer dependency, improving measurement reproducibility, and facilitating the scalability of vascular function testing in routine clinical practices. Furthermore, robust quality assurance and quality control frameworks, together with external validation systems, are needed to ensure the reliability and reproducibility of vascular function measurements, particularly in multicenter studies and large collaborative research networks. Harmonization of assessment protocols across institutions and the establishment of shared reference databases will be critical for enabling data integration, inter-study comparability, and large-scale collaborative investigations. Such efforts will also facilitate the development and validation of universally accepted diagnostic thresholds and phenotype definitions. Emerging digital health technologies offer additional opportunities to accelerate the clinical implementation of vascular phenotyping. Artificial intelligence–assisted image analysis, cloud-based data-sharing platforms, wearable devices, and remote monitoring systems have the potential to improve workflow efficiency, support longitudinal assessment of vascular health, and enable continuous integration of multimodal vascular data into clinical decision-making. Although these technologies remain at varying stages of development and clinical validation, they represent promising tools for advancing precision vascular medicine and facilitating the translation of vascular phenotyping from research settings to routine clinical care.

### Clinical trial strategies for blood pressure–independent vascular protection

Future clinical trials should be specifically designed to determine whether vascular protection beyond blood pressure reduction translates to improved cardiovascular outcomes. One promising approach is the use of mechanism-based enrichment trial designs, in which participants are selected according to predefined vascular phenotypes—such as increased arterial stiffness, endothelial dysfunction, or microvascular dysfunction—to enrich for biological responsiveness and improve the ability to detect vascular-specific therapeutic effects independent of blood pressure lowering. In addition to conventional blood pressure reduction, vascular function–specific endpoints should be incorporated as complementary efficacy measures. These include PWV, FMD, RHI, augmentation index, indices of microvascular function, and imaging-based measures of vascular remodeling. Such endpoints may provide a more comprehensive assessment of treatment effects on vascular biology and facilitate evaluation of disease-modifying interventions. Advanced statistical approaches, including mediation analysis, should also be incorporated to clarify the causal pathways linking therapeutic interventions to cardiovascular outcomes. By quantifying the proportion of treatment benefits mediated through improvements in vascular function rather than blood pressure reduction alone, these analyses may help establish vascular function as a mechanistic therapeutic target rather than merely a surrogate biomarker. Biomarker-guided trial designs represent another promising strategy, whereby circulating biomarkers and multimodal vascular imaging are incorporated for patient stratification, treatment allocation, and assessment of therapeutic response. Such approaches may improve patient selection, increase trial efficiency, and facilitate the development of precision medicine strategies based on individual vascular biology. Finally, adequately powered head-to-head randomized controlled trials comparing antihypertensive therapies that achieve comparable blood pressure reduction but differ in their vascular biological effects will be essential. These studies would provide the most rigorous evidence to determine whether specific therapeutic interventions confer clinically meaningful vascular protection beyond blood pressure lowering and whether such benefits ultimately translate into superior cardiovascular outcomes.

### Feasibility and scalable implementation of vascular function assessment

The widespread implementation of comprehensive vascular function assessment remains challenging in routine clinical practice because of limitations in equipment availability, technical expertise, time, and cost of the procedure. Although advanced vascular phenotyping techniques—including arterial stiffness, endothelial function testing, and multimodality vascular imaging—provide valuable pathophysiological information, their routine application is currently limited in many healthcare settings. A pragmatic, stepwise implementation strategy (Fig. 3) may facilitate the gradual integration of vascular phenotyping into clinical practice. In the initial phase, readily available clinical parameters and surrogate markers, including blood pressure variability, pulse pressure, ankle–brachial index, and routinely measured laboratory biomarkers, may be used to provide an initial estimate of vascular risk and guide patient selection. In the subsequent phase, more specialized vascular function assessments may be selectively performed in patients at high cardiovascular risk, those with resistant or difficult-to-control hypertension, or individuals in whom additional phenotypic characterization is expected to influence therapeutic decision making. Broader implementation will depend on continued improvements in affordability, simplification of measurement protocols, automation of data acquisition and image analysis, and seamless integration with digital health platforms, including digital hypertension management systems. Furthermore, advances in portable diagnostic technologies, AI–assisted image interpretation, wearable sensors, and remote monitoring platforms are expected to improve accessibility, reduce operational burden, and enable scalable deployment of vascular function assessment across diverse clinical settings. Such a tiered implementation model has the potential to balance clinical feasibility with diagnostic precision, thereby facilitating the translation of vascular phenotyping from specialized research centers to routine cardiovascular care. Ultimately, the scalable implementation of vascular function assessment will be an essential step toward the realization of precision vascular medicine.

### Positioning a vessel-centric framework within current hypertension guidelines

Current major hypertension guidelines, including those developed by the American College of Cardiology/American Heart Association, the European Society of Cardiology/European Society of Hypertension, and the Japanese Society of Hypertension [1–4], primarily recommend treatment strategies based on blood pressure levels, global cardiovascular risk assessment, hypertension-mediated organ damage, and the presence of comorbid conditions. Although these evidence-based approaches have substantially improved cardiovascular outcomes, they incorporate direct assessments of vascular biology only to a limited extent. The proposed vessel-centric framework is intended to complement, rather than replace, existing guideline-directed management by incorporating vascular phenotyping into cardiovascular risk assessment and therapeutic decision making. This approach recognizes the biological heterogeneity of hypertension and emphasizes that distinct vascular phenotypes—including large artery stiffness, endothelial dysfunction, and microvascular disease—may contribute differentially to cardiovascular risk despite similar levels of blood pressure. Consequently, vascular characterization may refine risk stratification within existing guideline categories and facilitate more individualized, mechanism-based therapeutic selections. Importantly, the proposed framework should be regarded as an adjunctive strategy that builds on current evidence-based recommendations rather than an alternative treatment paradigm. Its primary objective is to integrate vascular biology into contemporary hypertension management while preserving the established principles of guideline-directed blood pressure control and comprehensive cardiovascular risk reduction. However, the clinical utility of this approach remains to be established. Prospective clinical studies are therefore required to determine whether incorporation of vascular phenotyping into guideline-based care improves risk prediction, optimizes treatment selection, and ultimately provides incremental cardiovascular benefits beyond current blood pressure- and risk-based management strategies. Successful validation of this concept could provide the foundation for future guideline recommendations incorporating mechanism-based vascular phenotyping into precision hypertension management.

## Conclusion

Hypertension is a systemic vascular wall disease with structural and functional abnormalities that sustain pressure elevation. Blood pressure reflects issues like endothelial dysfunction, redox imbalance, immune activation, microvascular remodeling, and arterial stiffening. Endothelial dysfunction reduces NO, promoting vasoconstriction, inflammation, and thrombosis. Oxidative stress worsens this via eNOS uncoupling and redox signaling. Immune activation, especially salt-sensitive and Th17 pathways, increases vascular inflammation and remodeling. Microvascular rarefaction raises resistance, while artery stiffening increases central pulsatile load, damaging organs. These mechanisms maintain hypertension and accelerate organ injury, even with controlled brachial blood pressure. A vessel-centric paradigm has implications. Risk stratification would include endothelial function, arterial stiffness, central hemodynamics, and microvascular integrity. Therapeutic evaluation would focus on vascular restoration alongside blood pressure reduction. Clinical management would shift to mechanism-guided intervention tailored to vascular phenotypes. This paradigm views blood pressure as one aspect of vascular health. Recognizing vascular function as central to hypertension and its complications enables earlier detection, precise therapy, and meaningful treatment success definitions. Restoring vascular homeostasis, not just normalizing brachial pressure, becomes the ultimate therapeutic goal, representing the next frontier in cardiovascular medicine.
